# The CINAMR (Clinical Information Network-Antimicrobial Resistance) Project: A pilot microbial surveillance using hospitals linked to regional laboratories in Kenya: Study Protocol

**DOI:** 10.12688/wellcomeopenres.18289.1

**Published:** 2022-10-12

**Authors:** Samuel Akech, Brian Nyamwaya, Jackline Gachoki, Morris Ogero, Joyce Kigo, Michuki Maina, Edna Mutua, Ednah Ooko, Philip Bejon, Salim Mwarumba, Felix Bahati, Benedict Mvera, Robert Musyimi, Robert Onsare, Jack Hutter, Emmanuel Tanui, Evelyn Wesangula, Paul Turner, Susanna Dunachie, Olivia Lucey, Jacob McKnight, CINAMR Investigators

**Affiliations:** 1 Kenya Medical Research Institute-Wellcome Trust Research Programme, Kilifi, Kenya; 2 Centre for Tropical Medicine and Global Health, Nuffield Department of Medicine, University of Oxford, Oxford, UK; 3 Kenya Medical Research Institute-Centre for Microbiology Research, Nairobi, Kenya; 4 United States Army Medical Research Directorate-Africa/Kenya (USAMRD-A/K), Kombewa, Kenya; 5 Kenya Ministry of Health - AMR National Secretariat, Nairobi, Kenya; 6 Cambodia Oxford Medical Research Unit (COMRU), Angkor Hospital for Children, Siem Reap, Cambodia; 7 Mahidol-Oxford Tropical Medicine Research Unit, University of Mahidol, Bangkok, Thailand; 8 Imperial College London, London, UK

**Keywords:** AMR, surveillance, protocol, bacterial, resistance, Kenya, Africa

## Abstract

**Background:** Antimicrobial resistance (AMR) is a global threat and is thought to be acute in low-and middle-income country (LMIC) settings, including in Kenya, but there is limited unbiased surveillance that can provide reliable estimates of its burden. Current efforts to build capacity for microbiology testing in Kenya are unlikely to result in systematic routine microbiological testing in the near term. Therefore, there is little prospect for microbiological support to inform clinical diagnoses nor for indicating the burden of AMR and for guiding empirical choice of antibiotics.

**Objective:** We aim to build on an existing collaboration, the Clinical Information Network (CIN), to pilot microbiological surveillance using a *‘hub-and-spoke’* model where selected hospitals are linked to high quality microbiology research laboratories.

**Methods:** Children admitted to paediatric wards of 12 participating hospitals will have a sample taken for blood culture at admission before antibiotics are started. Indication for blood culture will be a clinician’s prescription of antibiotics. Samples will then be transported daily to the research laboratories for culture and antibiotic susceptibility testing and results relayed back to clinicians for patient management. The surveillance will take place for 6 months in each hospital. Separately, we shall conduct semi-structured interviews with frontline health workers to explore the feasibility and utility of this approach. We will also seek to understand how the availability of microbiology results might inform antibiotic stewardship, and as an interim step to the development of better national or regional laboratories linked to routine surveillance.

**Conclusions:** If feasible, this approach is less costly and periodic *‘hub-and-spoke’* surveillance can be used to track AMR trends and to broadly guide empirical antibiotic guidance meaning it is likely to be more sustainable than establishing functional microbiological facilities in each hospital in a LMIC setting.

## Introduction

Antimicrobial resistance (AMR) is a major threat to human health and is thought to be particularly acute in low-and middle-income country (LMIC) settings where infectious disease is a prominent cause of morbidity and mortality and recent estimates show that AMR causes more deaths than HIV or malaria ^1, 2^. Possible causes of AMR include overuse, misuse, improper disposal, and counterfeiting of antimicrobials used in human health, livestock, or food production. Infection with resistant organisms results in many adverse consequences including increased risk of death, prolonged hospital stay, longer duration of illness, increased risk of mortality and morbidity in patients undergoing surgical and other invasive procedures, increased costs to the healthcare system, and increased costs to patients including loss of productivity from prolonged illness. AMR has been recognized as a global problem culminating in the development of the Global Action Plan to Combat AMR in 2015 following a request by the World Health Assembly in resolution WHA 67.25 in May 2014. This was preceded by the Global Strategy for Containment of AMR issued by WHO in 2001 and several resolutions approved by WHO Member States to address the strategy’s objectives. The global plan recommends development of national action plans and Kenya has developed a National Action Plan to combat AMR (NAP) for the period 2017–2022. Kenya’s NAP, like the global action plan, has five strategic objectives and the second objective is to ‘strengthen knowledge and evidence base through surveillance and research’. A situation analysis done in Kenya in 2016 showed an increase in AMR, but the rate of increase remains unknown and there is a lack of systematic AMR surveillance. Many countries in Africa rely on estimates of AMR from a few centres and reports of high levels of resistance are uncertain ^3^.

AMR surveillance is crucial for informing policies and infection prevention and control (IPC) responses, assessment for the spread of AMR, measuring impact of AMR, and to guide interventions. However, surveillance data for AMR in most LMIC countries, including Kenya, have been limited to tertiary referral private hospitals, or research facilities. Data from tertiary referral or private hospitals are likely to be biased towards complex infections, treatment failures, and hospital-acquired infection. Data from research facilities are too sparse to ensure generalizability and most tend to focus on specific clinical questions or syndromes, for example, to describe existence of specific resistant isolates or are focused on isolates ^4– 17^. Therefore, existing surveillance do not provide insight into the cause, type, scale, or spread of antimicrobial resistance.

There have been efforts aimed at strengthening the AMR surveillance system in Kenya supported by several partners ^18^ including The Fleming Fund, the East Africa Public Health Laboratory Networking Project, Infectious Disease Detection and Surveillance (IDDS), and The Medicines, Technologies, and Pharmaceutical Services (MTaPS) Program that have focused on building laboratory capacity in selected hospitals, but these efforts may only result in passive, non-systematic (leading to unclear denominators), laboratory/ isolate-based surveillance and produce AMR data that will not describe the burden of AMR and is less useful in informing empirical choice of antibiotic treatment for guidance development. There are also concerns that hospitals may be unable to retain highly skilled staff trained in microbiology through these initiatives and hospitals’ capabilities to sustain microbiological services beyond the life of these projects are yet to be established ^4^.

Establishing an effective routine AMR surveillance is complex and require a functional laboratory that can perform culture, identification of pathogens and drug susceptibility testing using appropriate techniques, and procedures for external quality assurance (EQA) and is likely to remain a challenge to establish in many hospitals ^4, 18, 19^. Kenya enrolled into the World Health Organization (WHO) led Global Antimicrobial Resistance Surveillance System (GLASS) in May 2016. In the 2017 GLASS report, Kenya reported having established eight surveillance sites, eight hospitals and eight outpatient clinics, but did not submit 2016 data at the end of the data call. The 2020 GLASS report (which includes data submitted by 31 ^st^ July 2019), showed that Kenya reported only information on the national surveillance system with no specific AMR data. It also reported that eight surveillance sites (four hospital and four outpatient) and four laboratories were performing antimicrobial susceptibility testing (AST) with external quality assurance (EQA) provided to these laboratories for bacterial identification and AST for all GLASS pathogens.

This study aims to build on an existing collaboration, the Clinical Information Network (CIN), described below, to pilot microbiological surveillance using a *“hub-and-spoke”* model with high quality regional microbiology research laboratories linked to multiple hospitals, which could be scalable in an LMIC setting. The focus is on six bacterial bloodstream pathogens prioritized by GLASS for surveillance including *Escherichia coli, Klebsiella pneumoniae, Acinetobacter baumannii, Staphylococcus aureus, Streptococcus pneumoniae, Salmonella* spp. This study also explores the feasibility, and utility of periodic surveillance sampling across multiple public hospitals to inform antibiotic stewardship, monitor trends in AMR, and as an interim step to development of better national laboratories linked to routine surveillance. We also seek to understand how the microbiology data is used in hospitals and if the findings can inform national, regional, and local formularies and guidelines. Finally, we seek to determine if this model could be used over the long term and expanded in Kenya or the region.

## Protocol

### Ethics

The study was reviewed and approved by the Kenya Medical Research Institute Scientific and Ethics Review Unit (KEMRI SERU), Walter Reed Army Research Institute’s Institutional Review Board (WRAIR IRB), and Oxford Tropical Research Ethics Committee (OxTREC). Approval for waiver of consent was obtained as blood cultures are deemed routine and the microbiology results from the study are used for clinical management of individual patients-SERU ethical approval on August 10, 2021.

### Study site

KEMRI-Wellcome Trust Research Programme (KWTRP) in partnership with the Kenya Paediatric Association/Kenya Paediatric Research Consortium (KPA/KEPRECON), Kenya’s Ministry of Health and selected County Referral Hospitals in Kenya have developed a collaboration since 2013 referred to as the Clinical Information Network (CIN) ^20, 21^. CIN seeks to improve patient care, data quality and reporting, and strengthen in-patient paediatric surveillance data by strengthening collection and use of routine data through audit and feedback. CIN now covers 24 hospitals across 19 counties and captures data on clinical admission details including history at admission, admission examination findings, admission treatments, investigations, diagnoses, and outcomes for all children aged 1 month and older admitted to paediatric wards ^21^. The typical upper age limit for admissions to the paediatric wards is 12 years, but a few hospitals may admit older children.

The hospitals have adopted uniform standardised medical records for paediatric and neonatal admissions that have been developed through consultative processes convened by KPA. These forms are printed by the hospitals and are part of the official patient medical file and remain the property of the hospital and not the research partners. Clinical care is provided by clinical staff employed by the hospitals. The only personnel based at the hospital employed by the research project is a single data clerk. The single clerk enters data into a REDCap database and the hospital owns the data. A copy of the data is shared with KWTRP by syncing into a server based at KWTRP offices in Nairobi. Auto-generated R scripts are used to prepare quarterly feedback reports for each hospital on processes of care for common conditions using the data shared at the KWTRP server. Hospital paediatricians/lead clinicians share these reports with their teams and identify any areas that may require improvement. Data captured by the CIN data clerk includes minimal data needed for CINAMR including date, age, gender, clinical syndrome, co-morbidities, and antibiotics prescribed. CIN database has fields for entering microbiology results, but these are often empty because of little microbiology testing at these hospitals and the same CIN data clerk will enter microbiology results.

The CIN project is coordinated by a clinical coordinator, typically a senior clinician, and a central data management team. Patient management in the hospitals is largely syndrome-based in accordance with the Kenyan Paediatric guidelines, which are adapted from WHO guidance ^22^, as previously described ^21^. Malaria testing by hospital laboratories and rapid HIV testing are done in all hospitals, and GeneXpert MTB/RIF assay is available although not optimally used. Antibiotic prescription in these hospitals, and hospitals widely in Kenya, is largely empirical and cultures (blood, urine, cerebrospinal fluid, stool) are rare. Microbiological diagnosis is hampered by structural deficits (lack of equipment, supplies, and adequate numbers of trained staff) and process deficits (lack of quality assurance for the tests/sample handling, lack of confidence in the laboratories amongst medical staff etc.). CINAMR is proposed to take place in hospitals that are currently part of CIN or that have expressed willingness or are in the process of joining CIN. The hospitals selected will be those where there are no current competing AMR projects, willing to participate in the project, have no existing capacity to perform microbiology culture in their laboratories. The surveillance will be conducted in paediatric wards in up to 12 public level 4–5 (first referral level) hospitals in Kenya.

### Inclusion criteria

All children where antibiotic prescription is made at admission will be considered to have suspected community-acquired infection (CAI) and will be eligible for blood culture. Blood cultures in cases where antibiotics are changed or commenced after the first 72 hours from admission will be considered meant to investigate hospital acquired infection (HAI). HAI is not the focus of CINAMR, but such blood samples will be sent for culture in the same way as samples collected at admission and some of these may represent treatment failures.

### Exclusion criteria

Patients admitted outside the children’s ward.

### Sample size determination

We expect to obtain 5–6 blood cultures/hospital/weekday, for 6 months at each of 12 hospitals, leading to approximately 750 cultures/hospital and 9,000 cultures across 12 hospitals. Expected daily numbers are based on admission data from existing CIN hospitals. Expecting 8% of blood culture samples collected to be positive for a pathogen, will result in 60 positive blood cultures/hospital. This would give power for a 95% confidence interval (CI) for proportions of 0.37–0.63 around a hypothetical proportion of 0.5 resistant organisms among all positive cultures per hospital, 95% CI of 0.44–0.56 for a subnational grouping comprising 1/3 of the total, or 0.46–0.54 overall. We would thus have substantial power to determine the prevalence of resistance in sub-groups.

### Study procedures

The surveillance will run for 6 months in each hospital in two phases comprising six hospitals each. Blood culture samples are primarily to be collected by routine hospital clinical teams supported by a dedicated study staff (surveillance officer). The surveillance officer is a qualified laboratory technologist. The surveillance officers work with the hospital clinicians to identify patients with suspected bacterial infections (this is defined as any child who is started on antibiotics) at admission and collect a blood culture sample before antibiotics are commenced and in conformity with hospital clinical procedures. The surveillance officer also provides logistical support, and facilitates testing, and relays results to the clinical team. Blood culture (BC) packs including syringes, needles, sterile swabs, gloves, and BD BACTEC blood culture bottles are provided for the study period at each site. Surveillance officers and relevant hospital staff will be trained in blood culture sampling to ensure optimal yield and aseptic technique to limit sample contamination. The surveillance officer also coordinates transport of samples to the testing laboratory. Blood culture samples are couriered daily to the nearest laboratory. The laboratories are in three KEMRI centres distributed across Kenya: coast (Kilifi- KEMRI Centre for Geographic Medicine Research- Coast (CGMRC), the KEMRI Wellcome Trust Research Programme (KWTRP) laboratory), central (Nairobi-KEMRI Centre for Microbiology Research (CMR) Laboratory) and western (Kisumu/ Kombewa-US Army Medical Research Directorate-Africa/Kenya (USAMRD-A/K). Samples will be collected from hospitals Monday to Friday from 3–4pm and transported to respective laboratories. Sample collection for the surveillance will take place between 7am–3pm on weekdays. However, clinicians will be free to take blood samples for cultures during study hours for patients who are admitted outside the surveillance hours. In such cases of delayed sample collection, information collected on antibiotic use and duration since start of antibiotics will be useful in interpretation of results. Recent antibiotic treatment history, including any antibiotics currently used and duration since commencement of antibiotics will be captured at the time of blood collection and entered into the database.

Positive microbiology results will be fed back to surveillance officers (and clinicians) on the same working day of identification at each hospital. A final culture result will be sent after the 5-day culture, which would identify slow-growing organisms. Positive results sent to the hospitals are accompanied by an interpretation of implications for susceptibility results (sensitive, intermediate, and resistance) to antibiotic drugs as a guide to clinicians in making decisions on antibiotic treatments.

Hospitals will also receive regular monthly hospital-specific reports on pathogens isolated together with drug susceptibility patterns. Aggregate results broken down by hospital will be provided to the Ministry of Health (MoH) monthly and will be available to feed into MoH AMR surveillance policy discussions and development of national antibiotic guidelines. Data will also include percentage positivity of blood cultures per site and by age group with proportion of contaminants monthly.

### Sample transport to testing laboratories

Inoculated BC bottles are packaged according to international regulations adopted by the International Air Transportation Association (IATA) and transported at room temperature via courier daily to the designated automated blood culture system (BD BACTEC) located at the nearest laboratory. A maximum of 11 hours delay between sample collection at the hospitals and loading onto the blood culture system at the regional KEMRI laboratories may occur in a few cases, but studies suggest that up to 12 hours delay at room temperature does not compromise sample positivity yields ^23– 26^.

### Microbiology testing

On arrival at the respective KEMRI laboratory, the blood culture bottles received will be booked onto the electronic laboratory system and loaded onto the blood culture system for a 5-day incubation period. Blood cultures which flag positive are processed as per established laboratory standard operating procedures including Gram stain and subculture onto relevant agar with appropriate incubation. Microorganism identification is based on culture morphology, biochemical profiling (including automated methods) and use of matrix-assisted laser desorption/ionization (MALDI-TOF) mass spectrometry (in Kilifi). Culture-based phenotypic susceptibility testing is performed by disc diffusion, with zone sizes recorded and interpreted using current Clinical and Laboratory Standards Institute (CLSI) breakpoints with use of minimum inhibition concentrations (MICs) by E-test where necessary. Alternatively, automated susceptibility testing (BD Phoenix™) may be used. BC bottles will be discarded in accordance with the laboratories’ standard operating procedures after culturing. The three laboratories use the same version of CLSI for interpretation and microbiology testing methods coordinated from the KWTRP laboratory.

GLASS target organisms ( *E. coli, K. pneumoniae, A. baumannii, S. aureus, S. pneumoniae, Salmonella* spp) will be identified alongside other common causes of bacteraemia. Isolates of select organisms such as *S. pneumoniae*, *H. influenzae* and *Salmonella* may be further characterized through subtyping to identify organism-specific serotypes ^27– 29^. Serotyping will be performed at the KEMRI laboratories using standard approaches. Phenotypic sensitivity patterns (according to the latest CLSI guidelines) will be employed to identify common resistance mechanisms in both Gram-negative and Gram-positive organisms, with focus on antimicrobial sensitivity to first- and second-line treatment regimens for common clinical syndromes. Examples of resistance mechanisms include extended-spectrum (ESBL) or carbapenem-hydrolyzing beta-lactamases in Gram-negatives, penicillin resistant *Streptococcus pneumoniae*, methicillin resistant *S. aureus* (MRSA), and vancomycin resistant *Enterococci* (VRE). Aminoglycoside and fluoroquinolone resistance are incorporated for appropriate organisms. Drugs tested for the different bacteria-drug combinations are similar for the three laboratories and incorporate bacteria-drug combinations recommended in WHO guidelines.

Contaminants in this setting without central venous lines or ventilation such as coagulase-negative Staphylococci ^30^, *Bacillus* spp, *Micrococcus* spp, Coryneforms, viridans Streptococcus group, and non-baumannii *Acinetobacter* spp will be logged for quality control and feedback, but not tested for AMR or individually reported.

The three laboratories have good quality assurance measures in place. The KWTRP laboratory in Kilifi has implemented good clinical laboratory practice (GCLP), has quality assurance measures in place, and is accredited by Qualogy® ^31^. KEMRI-USAMRD-A/K (Kisumu/Kombewa) has well established GCLP compliant research laboratories with internal and external quality assurance programs and the KEMRI ISO team. KEMRI-USAMRD-A/K laboratory has service contracts for all equipment and calibrations are done annually between KEMRI USAMRD-A/K, Walter Reed Army Research Institute (WRAIR) maintenance team and/or its external vendors. KEMRI’s Centre for Microbiology Research (CMR) (Nairobi) also has quality assurance measures in place as a result of partnership with KWTRP laboratory ^32^.

### Archiving of isolates

Anonymized bacterial isolates will be sent to the KWTRP laboratory in Kilifi and stored for more detailed characterization of AMR mechanisms using various methods including MALDI-TOF and whole genome sequencing later.

### Health systems analysis

We aim to explore the potential use of surveillance data to inform national treatment policy development; how AMR data might influence local formularies and prescribing practices; and what might be the best mechanisms to use data going forward to achieve effective antimicrobial stewardship (e.g., through facility Medicines and Therapeutic Committees or Continuous Medical Education). We will also investigate how laboratories in public hospitals might become part of future surveillance networks. This qualitative health systems work is undertaken at national and county levels and in the same hospitals as the surveillance. Six hospitals are chosen to broadly represent the range of hospitals in Kenya considering infectious disease burden, rural versus urban locations, population served, size, ‘level’, and capacity. A minimum of five medical staff at each facility (and/or county) are interviewed, but this can be higher when more interviews are required to reach saturation.

Long form interviews are used for key informants at all levels of the health system. Those identified for interviews include national AMR stakeholders, laboratory technicians, pharmacists, clinicians, members of the Medicines and Therapeutics Committees (where they are functional), and members of the county medical teams involved in decision making regarding drug purchase and use. Analysis will be conducted of the current system that is used to choose and purchase antimicrobials at the county and hospital levels, and the prescribing systems that exist in Kenyan hospitals, inclusive of both formal guidance such as guidelines and formularies, but also informal mechanisms such as norms established by consultants and influential clinicians. We will also seek to understand how these systems and practices might be altered by the availability of data concerning AMR relevant to each facility studied. Interviews will also be done with relevant national stakeholders around plans for developing AMR capacity within the country. Interviews may be conducted online using smartphone-based applications or through phone calls where necessitated by COVID-19 mitigation measures. Participants are consented for the interviews and consent is provided digitally, through email, where interviews are done virtually. In all cases, the interviews will be audio-recorded for transcription purposes. Data analysis will involve creating a detailed description of the range of systems in place for making decisions regarding local, regional, and national drug use in Kenya and how these do or do not consider data on AMR.

### Data collection

The microbiology results will also be entered as part of routine medical records captured in CIN for participating hospitals. The central data management at KWTRP will work with the CIN data clerk based at the hospital to oversee data entry, error checking and reconciliation, cleaning, and storage. Existing data collection systems will be used at one hospital where KWTRP already runs an ongoing surveillance. De-identified CIN data shared with KWTRP and managed under appropriate research governance procedures. Anonymized individual level data will be shared with other AMR surveillance initiatives locally and internationally following approval from the KWTRP Data Governance Committee.

### Data analysis

Audio-recorded interviews that have been transcribed will be imported into NVIVO 10 software. Each transcript will have a unique identifier comprising hospital code, date, to enhance anonymity and facilitate informed analysis. Data analysis will involve creating a detailed description of the range of systems in place for making decisions regarding local, regional, and national drug use in Kenya and how these do or do not consider data on AMR. It is expected that this analysis will lead to exploratory discussion within the research team to inform future phases of the project.

Individual results will be fed back to responsible clinicians and hospitals will also receive regular monthly hospital-specific reports on pathogens isolated together with drug susceptibility patterns. Aggregate results broken down by hospital will be provided to the Ministry of Health (MoH) monthly and will be available to feed into MoH AMR surveillance policy discussions and development of national antibiotic guidelines. Data will also include percentage positivity of blood cultures per site and by age group with proportion of contaminants monthly. Anonymised patient level data including demographics, blood culture results, admission clinical characteristics, and hospital outcomes will used to describe the prevalence and burden on AMR in Kenya and anonymised data will also be shared with global initiatives to contribute to describing the global burden of AMR.

## Discussion

This study pilots a novel *‘hub-and spoke’* method for microbial surveillance where research laboratories with capacity to perform high quality microbiology process samples for nearby public hospitals. The study also explores the feasibility and acceptability of the surveillance approach while also investigating how availability of microbiology data might inform clinical behaviour and antibiotic stewardship efforts. The surveillance approach is systematic and involves collecting samples for blood culture in all children where antibiotics are prescribed at the time of admission to hospital. There is also linkage between clinical and laboratory surveillance and the culture data will be linked to clinical outcomes and this will be useful in informing the burden of bacterial bloodstream infections and AMR. A recent systematic analysis of the global burden has shown that AMR causes more deaths compared to malaria or HIV and causes nearly four times more deaths in Africa compared to the region with lowest case-fatality ^2^. However, this analysis also pointed out the scarcity of microbiology data linked to clinical outcomes, possible unrepresentative data, and uncertainty in antibiotics use and availability amongst other reasons that make uncertain estimates for LMICs, especially sub-Saharan Africa.

The CINAMR project does not seek to duplicate past or existing laboratory capacity building initiatives in Kenya, which have had limited success in achieving sustained use of diagnostic testing for microbiology ^4, 18, 19^ and limited data to support national AMR surveillance. Systematic surveillance with a broad pragmatic inclusion criterion will result in data that is representative of community acquired bloodstream bacterial infections. The microbiology surveillance will be linked to CIN and microbiology data collected as part of routine medical records into the CIN database, therefore, in-hospital clinical outcomes will be available. It may be argued that using administration/prescription of antibiotics at admission as an entry criterion for blood cultures is not clinically intuitive, resulting in many blood cultures that would otherwise not be ordered by clinicians. However, this criterion has been used in many surveillance efforts since it is an objective indication that the clinician suspects a bacterial infection. Prior use of antibiotics may lower the yield of blood cultures in this study and result in inaccurately low estimates of community acquired bacterial blood stream infections. History of antibiotic use is recorded and is used in interpretation of blood culture results and will also be used in wider interpretation of results. The CINAMR study does not have follow-up of participants post discharge and this may be seen as a limitation in the estimation of the burden of AMR, but this is not the focus of CINAMR, which seeks to demonstrate the feasibility of the *‘hub-and spoke’* approach.

In summary CINAMR will demonstrate the feasibility and acceptability of the *‘hub-and spoke’* approach to microbiology surveillance, while also generating data on community acquired bloodstream bacterial infections in Kenya. If this pilot demonstrates success, then this approach may have utility in periodic monitoring of development of AMR, in development of empirical antibiotic treatment guidelines, and possible avenues for hospitals to link to research laboratories for routine microbiology diagnosis without the need to establish their own functional microbiology services.

## Study status

Surveillance in the first set of hospitals commenced on January 24 ^th^, 2022. All 12 hospitals were enrolled by August 2022.

## Ethics approval

KEMRI Scientific and Ethical Review Committee approved the CINAMR study (SERU #4246), Oxford Tropical Research Ethics Committee (#36-21), and the Walter Reed Army Institute of Research HSPB and WRAIR IRB.

## Data Availability

No data are associated with this article. Ambrose Agweyu, Mike English, Jalemba Aluvaala, Caroline Tigoi, Wangeci Kagucia, Morris Ogero, Cynthia Khazenzi, Monica Musa, Joyce Kigo, Stephen Kamau, Jay Berkley, Anthony Scott, Nathanial Copeland, Lucas Tina, Samuel Kariuki, Lillian Musila, Linnet Munyasia, John Owino, Moses Inyanje, Violet Kitsao, Benson Murega, Fatihya Swaleh, Martin Maitima, Frederick Apollo, Patrick Kamau, Kenneth Muchiri, Joseph Kimani, Emma Namulala, Meshack Liru, Vitalis Juma, Boniface Nyumbile, Roselyne Malangachi, Grace Ochieng, Lydia Thuranira, Sylvia Mwathi, Grace Akech Ochieng, Elizabeth Jowi, David Kimutai, Christine Manyasi, Adem Achieng, Agnes Mithamo, Mwangi Wagura, Mercy Kamau, Yusuf Rasheed, Silvano Katayi, Wycliffe Dunde, Laurine Mukopi, Babra Murila, Cynthia Nduta, Serah Gathu, Andrew Odingo, Marion Ong’ayo, Sarah Kibira, Francis Wachiuri, Margaret Maina.
