# Supplementary material for: The CINAMR (Clinical Information Network-Antimicrobial Resistance) Project: A pilot microbial surveillance using hospitals linked to regional laboratories in Kenya: Study Protocol
Source: Wellcome Open Res. 2022 Oct 12;7:256. [Version 1] doi: 10.12688/wellcomeopenres.18289.1 (PMC10541537; doi:10.12688/wellcomeopenres.18289.1)
Supplement: Peer review history

# Reviewer report: Reviewer response for version 1

**DOI:** 10.21956/wellcomeopenres.20274.r67302

**Published:** 2023-09-29

**Authors:** Raphael Zozimus Sangeda

**Affiliations:** 1 Muhimbili University of Health and Allied Sciences, Dar es Salaam, Tanzania

The study aims to leverage an existing collaboration called the Clinical Information Network (CIN) to pilot a 'hub-and-spoke' model for microbiological surveillance. This model connects a selection of hospitals to high-quality research laboratories, allowing for the collection of blood culture samples from children admitted to these hospitals before antibiotic treatment. The samples are tested for bacterial cultures and antibiotic susceptibility, with results shared promptly with clinicians for patient care. This surveillance is supposed to run for six months in each hospital, and interviews with healthcare workers will assess the approach's feasibility and utility. 

 I agree that this strategy may offer a cost-effective and sustainable means of tracking AMR trends and guiding empirical antibiotic treatment in low- and middle-income settings, avoiding the need for establishing microbiological facilities in every hospital.

 The article may be approved.

Is the study design appropriate for the research question?

Yes

Is the rationale for, and objectives of, the study clearly described?

Yes

Are sufficient details of the methods provided to allow replication by others?

Yes

Are the datasets clearly presented in a useable and accessible format?

Not applicable

Reviewer Expertise:

Antimicrobial stewardship

I confirm that I have read this submission and believe that I have an appropriate level of expertise to confirm that it is of an acceptable scientific standard.

# Reviewer report: Reviewer response for version 1

**DOI:** 10.21956/wellcomeopenres.20274.r65397

**Published:** 2023-09-25

**Authors:** Huifang Wang

**Affiliations:** 1 Aix-Marseille Universite, Marseille, Provence-Alpes-Côte d'Azur, France

This study protocol describes a clinical information network-antimicrobial resistance project. They proposed to build microbiological surveillance using a "hub-and-spoke" model, where selected hospitals are linked to high quality microbiology research. This study will explore the feasibility of this model. Furthermore, they may compare the data quality, cost and diagnosis efficiency compared with establishing  functional microbiological facilities in each hospital in a low-and middle-income country.

 But I don't know which research question they could address here. If the feasibility of a surveillance model is part of the research question, thus I answered "partly" to the second question.

Is the study design appropriate for the research question?

Partly

Is the rationale for, and objectives of, the study clearly described?

Yes

Are sufficient details of the methods provided to allow replication by others?

Yes

Are the datasets clearly presented in a useable and accessible format?

Not applicable

Reviewer Expertise:

I am computational neuroscientists who works on the brain modelling and data analysis for brain disorders.

I confirm that I have read this submission and believe that I have an appropriate level of expertise to confirm that it is of an acceptable scientific standard.
